# Working memory capacity estimates moderate value learning for outcome-irrelevant features

**DOI:** 10.1038/s41598-022-21832-x

**Published:** 2022-11-16

**Authors:** Ido Ben-Artzi, Roy Luria, Nitzan Shahar

**Affiliations:** 1 School of Psychological Sciences, Tel Aviv University, Tel Aviv, Israel; 2 Sagol School of Neuroscience, Tel Aviv University, Tel Aviv, Israel

**Keywords:** Reward, Working memory, Cognitive control, Human behaviour

## Abstract

To establish accurate action-outcome associations in the environment, individuals must refrain from assigning value to outcome-irrelevant features. However, studies have largely ignored the role of attentional control processes on action value updating. In the current study, we examined the extent to which working memory—a system that can filter and block the processing of irrelevant information in one’s mind—also filters outcome-irrelevant information during value-based learning. For this aim, 174 individuals completed a well-established working memory capacity measurement and a reinforcement learning task designed to estimate outcome-irrelevant learning. We replicated previous studies showing a group-level tendency to assign value to tasks’ response keys, despite clear instructions and practice suggesting they are irrelevant to the prediction of monetary outcomes. Importantly, individuals with higher working memory capacity were less likely to assign value to the outcome-irrelevant response keys, thus suggesting a significant moderation effect of working memory capacity on outcome-irrelevant learning. We discuss the role of working memory processing on value-based learning through the lens of a cognitive control failure.

## Introduction

Throughout their lives, individuals infer causal associations between actions and outcomes as they experience and navigate dynamic and complex environments. Importantly, forming these associations requires cognitive flexibility and resources. Most environments are feature-rich, thus requiring the human agent to filter out irrelevant information when forming action-outcome associations in their minds^1–3^. For example, think of a child considering whether to eat an apple or a pear. The visual and tactile cues of each fruit should predict its taste, whereas its position on a table and the hand with which it is taken should be deemed irrelevant and thus not assigned value nor be considered in the child’s choice of fruit.

Reinforcement learning studies have described in depth individuals' tendency to learn action-value associations in a trial-and-error manner^4–6^. However, most of these studies disregarded the influence of outcome-irrelevant information on value-based learning. Recently, new evidence has emerged suggesting individuals do attribute value to outcome-irrelevant features of an action, even when they hold certain and explicit knowledge that those features have no predictive value for the outcome^7–9^. Specifically, in recent studies participants were required to make choices to gain monetary rewards while some aspects of the task had no causal association with the delivery of the reward. Participants were encouraged using both explicit instructions and prolonged training to ignore outcome-irrelevant aspects when making value-based choices. Yet, the evidence clearly suggested individuals engaged in outcome-irrelevant learning, defined as a tendency to assign value to features of the environment that are known to the individual with high certainty as holding no causal association with an adjacent outcome^7–9^. A fundamental question thus remains regarding the cognitive mechanisms that allow the human agent to refrain from assigning value to features of the environment that are known as having no (or little) causal association with an outcome.

Outside the realm of reinforcement learning and value-based learning, attentional control studies have extensively examined the influence of irrelevant information on individuals’ choices^10–12^. Specifically, a well-known attentional control system mostly studied outside the context of value-based learning is working memory. Working memory processing is known to reduce attention to irrelevant features including, for example, shape, color, or location of target stimuli^13–15^. Studies have systematically shown that even when observers have explicit and certain knowledge regarding the irrelevance of particular characteristics of a stimulus, attention regulation processes remain imperfect, resulting in a consistent influence of irrelevant information on decision-making^16,17^. A distinct feature of working memory is its limited capacity^18–20^. Previous studies have highlighted individual differences in working memory capacity, demonstrating that lower capacity is associated with a reduced ability to filter out irrelevant information^21–25^.

Since working memory processing has been mostly studied outside the context of value-based decision-making^16,26^, it is unknown whether the same working memory mechanisms that filter task-irrelevant information (e.g., distracting stimuli), are also engaged in the filtering of outcome-irrelevant information during value-based learning^5^. Therefore, in the current study, we examined whether working memory resources are required to refrain from assigning credit to outcome-irrelevant information. Considering findings from the attentional control literature, we further examined whether individuals with low, versus high, working memory capacity will be less capable to withhold credit from being assigned to outcome-irrelevant information, especially when it is relevant to an ongoing task^13,16,27^. It should be noted that some studies addressed working memory in the context of reinforcement learning as a one-shot learning system that perfectly retains recent action-outcome associations^28–31^. However, here we mostly follow the definition of working memory as an attentional system that enables the filtering of irrelevant information^12,22–25^.

In this pre-registered study, individuals performed a card game in which they were asked to choose cards to gain a monetary reward (i.e., multiple armed bandit task). Our task design allowed us to disentangle and estimate value updating to the outcome-irrelevant task features, since only cards, but not their randomly assigned response keys, predicted the monetary outcome. Furthermore, we included visual working memory encoding and retrieval phases with varying degrees of load in between trials of the reinforcement learning card task in order to examine the influence of visual working memory load on outcome-irrelevant learning. Finally, we estimated individuals’ working memory capacity using a well-known change-detection task^20,32^. Our results replicated previous studies by demonstrating a robust group-level effect of outcome-irrelevant learning. Moreover, we found significant individual differences such that only 55% of individuals demonstrated outcome-irrelevant learning. Importantly, working memory capacity showed a substantial moderation effect, such that reward had a lower influence on the selection of outcome-irrelevant response keys for high versus low-capacity individuals. However, to our surprise, the within-task working memory load manipulation did not influence outcome-irrelevant learning. We discuss these results by addressing the strengths and limitations of findings from reinforcement learning and attentional control literature, as well as the recent theoretical integration of the two perspectives.

## Methods

### Participants

174 Prolific workers (age mean = 27.1, range 18–49; 80 males, 93 females, 1 other) completed three online sessions across three consecutive days in return for monetary compensation (see SI). All participants reported normal or corrected vision, and no current or past psychiatric or neurological diagnosis (see SI). The study protocol was approved by the Research Ethics Council of Tel-Aviv University and all participants signed informed consent before participating in the study. All methods were carried out in accordance with relevant named guidelines and regulations.

### Procedure

In the first session, 200 participants performed a working memory capacity measurement. In the second and third sessions 178/174 participants respectively, completed a reinforcement learning task under three working memory load conditions (i.e., no-load, low-load, and high-load).

### Reinforcement learning task

**Figure 1:**
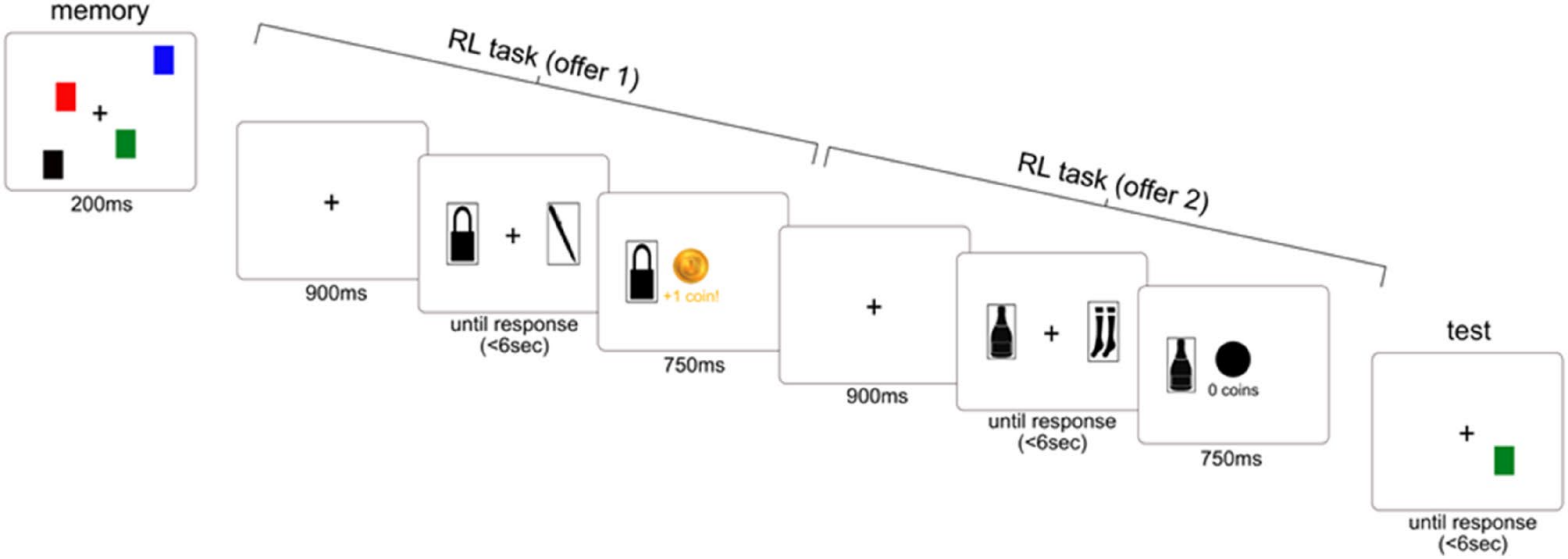
Trial sequence in the reinforcement learning task, which was performed under varying working memory loads. Participants were asked to first memorize a visual array (memory array stage), then make two choices across two card offers (reinforcement learning bandit task stage), and finally report whether a target was the same or different compared to the visual array that was memorized at the trial initiation (test array stage). Working memory load was manipulated between blocks by including in the memory stage either four random colored squares (high load), one random colored square (low load), or a fixed color square throughout the entire block, thus not requiring subjects to maintain the color in working memory during the task (no-load).

Participants completed a reinforcement learning bandit task interleaved between the memory array and test array stages of a working memory task (Fig. 1). This design allowed us to examine outcome-irrelevant learning processes under different working memory load conditions. Trials started with a memory array stage where participants had to memorize a visual array (i.e., colored squares). Next, participants made two decisions on two sequential offers of a reinforcement learning bandit task where they had to choose one of two cards to gain monetary rewards. Finally, the trial ended with a test array stage, where participants were asked to report whether a newly presented colored square was part of the initial visual array or not. We will now describe the memory array, multi-armed bandit trials, and test array stages that were included in each trial in more detail:

The memory array stage of the working memory task included the brief presentation (200 ms) of a visual array (i.e., colored squares) which the participants had to remember until the test array stage. To manipulate working memory load, we included three types of visual arrays (see Fig. S1) ; (a) no-load (a fixed color for the entire block of trials), (b) low load (one colored square, with its color being randomly selected each trial by the computer), and (c) high load (four colored squares, with all colors being randomly selected by the computer each trial without replacement out of nine possible colors). The three conditions (i.e., no load, low and high load) were manipulated between blocks. The locations of the squares was further randomized in each trial by the computer (see SI for further details).

Reinforcement learning bandit task included two offers for each trial (interleaved between memory array and test array stages), which were designed to allow us to estimate credit assignment to the outcome-irrelevant response keys. In each trial, the computer allocated four cards (without return) to the two offers (i.e., first or second). Therefore, each offer allowed individuals to choose one of two cards, and these two cards were further randomly allocated to the right or left sides of the screen (see Fig. 1). Offers in the reinforcement learning bandit task started with a fixation (900 ms), followed by the presentation of two cards in the right/left location. Participants then chose a card freely using a right/left corresponding response-key press (‘s’ or ‘k’ keys in a QWERTY keyboard; until-response with a 6 s deadline). After making a choice the unchosen card disappeared and the chosen card remained to allow choice feedback (500 ms). Cards led probabilistically to reward (£0 or £1, play-pound coins; outcome presented for 750 ms) according to a true expected value (which slowly drifted across trials according to a predefined random walk; see SI). Participants were asked to do their best to make choices that will maximize monetary return. To further ensure participants’ motivation, we rewarded them with a monetary payment bonus at the end of the study according to their gains in the task. Importantly, only the cards predicted reward, but not locations or response-keys used to report cards’ selection. This fact is important since it renders any credit assignment to the location/response key as outcome-irrelevant learning^8,9^. Participants were told explicitly during the instruction phase that only cards predicted reward and not the response-keys used for their selection (see SI). Before starting the task, participants were asked to complete a short quiz (9 questions long) to ensure they read and understood the instructions (see SI), which also included a specific question regarding which task features predict a monetary outcome. Participants had to show 100% accuracy in the quiz, and if they scored lower, they were prompted to the beginning of the instructions phase and were asked to retake the quiz.

Test array stage included a target screen in which one colored square was randomly selected by the computer in a color that was either the ‘same’ or ‘different’ color as the square that appeared in the same location in the memory array stage. Participants were asked to respond ‘same’ or ‘different’ by pressing ‘s’ or ‘k’ response-keys (keyboard mapping was counterbalanced between participants; until-response with a 6 s deadline). Following the target screen, participants were presented with feedback indicating whether their response was correct or incorrect, which was then followed by a fixation screen (inter-trial interval, 500 ms fixation).

Across two sessions participants completed a total of 6 blocks of the reinforcement learning task. Each block included a different set of cards and had 50 trials. Participants received a monetary bonus at the end of the task based on their performance.

### Working memory capacity task

To measure working memory capacity participants were asked to maintain and retrieve visual information^20,32,33^. On each trial, a visual array of colored squares appeared (set-size of 4 or 8 squares; see SI). Squares in each array had distinct colors and were evenly spread across the screen. Each trial started with the presentation of the squares array (i.e., memory array phase, 200 ms), followed by a fixation cross (i.e., retention phase, 900 ms) and then a target (i.e., test array phase, until-response with a 6 s deadline). The target screen included one colored square randomly selected by the computer in a color that was either the ‘same’ or ‘different’ color as the square that appeared in the same location in the memorized array. Participants were asked to respond ‘same’ or ‘different’ by pressing ‘s’ or ‘k’ response-keys (keyboard mapping was counterbalanced between participants). Following the target screen participants saw feedback indicating whether their response was correct or incorrect, followed by a fixation screen (inter-trial interval, 500 ms fixation). Each participant completed 120 trials aimed to identify individual differences in working memory capacity. After data collection, we discovered that due to a technical error one location for target squares on the lower left side of the screen was never probed during the retrieval phase of the change detection task. Further analysis suggested this did not have an impact on our overall conclusion (see SI for further details).

### Estimating outcome-irrelevant learning

**Figure 2:**
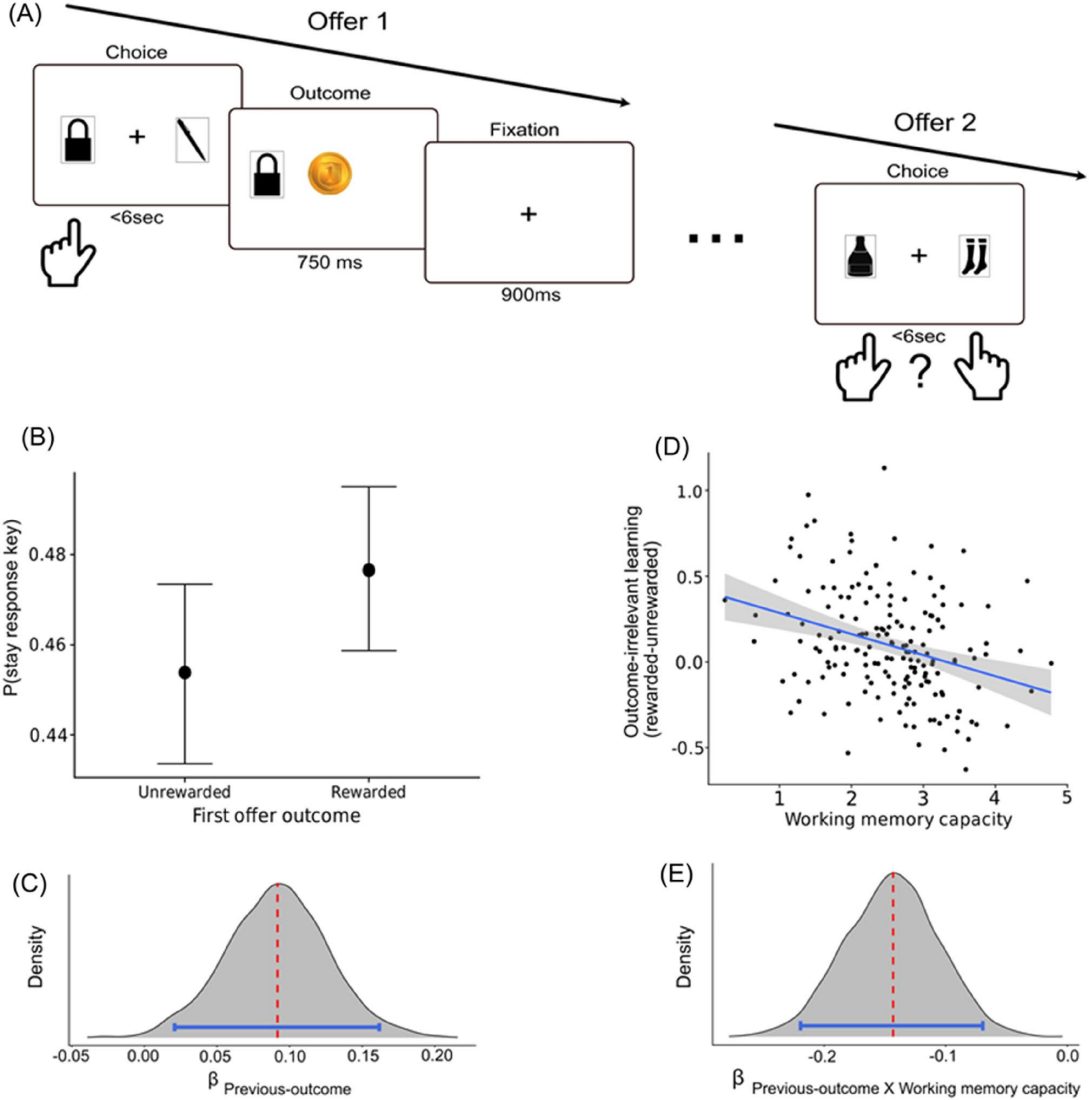
(**A**) Outcome-irrelevant learning was estimated as a tendency to stay with the previous response key selection as a function of previous-outcome (i.e., £0 vs £1), despite explicit knowledge that response keys did not predict outcomes in the task. (**B**) Marginal means estimates for response key stay probability show a greater stay tendency after reward vs. unrewarded trial, reflecting group level outcome-irrelevant learning (error bars represent HDI_95%_). (**C**) Posterior distribution for the regression coefficient of previous-outcome effect on response key stay probability describing group level outcome-irrelevant learning (red/blue lines indicate median/HDI_95%_, respectively). (**D**) Outcome-irrelevant learning differed substantially between individuals and was moderated by working memory capacity estimates. (**E**) Posterior distribution for the regression coefficient indicating the moderation effect (i.e., paired interaction) between previous-outcome and working memory capacity (red line showing the posterior median and blue lines the HDI_95%_).

To estimate outcome-irrelevant learning, we examined whether the outcome in the first offer (i.e. £0 vs. £1) affected response key selection in the second offer (see Fig. 2A). Specifically, we reasoned that credit will be assigned not only to the chosen card but also to a response key used for its selection [8,9]. This would mean that after a reward (i.e., £1) was obtained in the first offer, participants should be more likely to stay and choose the second offer with the same response key that was selected in the first one. However, if the choice in the first offer was unrewarded, participants should be more likely to switch their response key selection in the second offer. For example, assume that in the first offer the participant selected card A with a left response key, and observed a reward (£1). The left response key is now assumed to be more valuable in the individual’s mind, making it more likely that the left response key will be used in the second offer, where a different set of cards was offered. Therefore, outcome-irrelevant learning was assessed by estimating a regression parameter coefficient in a hierarchical Bayesian logistic regression, where repetition of response key selection from the first to the second offer (i.e., 0 vs. 1; Stay_response key_) was predicted using the outcome of the first offer (i.e., previous outcome, £0 vs. £1; Fig. 2B). The positive influence of previous-outcome on Stay_response key_ was considered as evidence for value assignment to the response key (i.e., outcome-irrelevant learning).

### Estimating working memory capacity

We calculated the detection accuracy in the visual working memory task (where reinforcement learning trials were not included) for each individual in each set-size condition (8 or 4 squares) to estimate working memory capacity. In each condition, capacity was calculated according to K = set_size ⋅ (2 ⋅ accuracy-1), and the average between the two conditions was used as a measure of overall capacity^34^. In our single-probe task, this is equivalent to a calculation based on hit and false alarm ratios^32^.

### Regression analysis

Hierarchical Bayesian regression analysis was performed using the ‘brms’ and ‘rstan’ R packages^35,36^. To examine posterior distributions we report 95% high-density interval (HDI) and probability of direction (pd). For all predictors, we used a pre-registered weakly informative prior, performed prior and posterior predictive checks, and verified that the conclusion did not change when using either wider or narrower priors (see SI). Bayes factors were calculated using the ‘bayestestR’ R package for both the null point estimate and the region of practical equivalence (ROPE)^37^. We used a ROPE interval in line with recent guidelines in bayesian statistics recommending supplementing a null-point Bayes factor estimate, with a more conservative Bayes factor calculating the relative likelihood of the effect being within the ROPE (theoretically negligible) vs. outside the ROPE^38^. We set the ROPE interval to be − 0.013 to 0.013 based on the rationale that a previous-outcome effect size (cohen’s d) of 0.007 or lower on the probability to stay with the same response key should be considered negligible.

### Data treatment

The first trial on each block and trials with implausibly quick reaction times (< 200 ms), or exceptionally slow reaction times (> 4000 ms) were omitted (5.52% of all trials). We omitted participants for the following reasons: (a) Participants with a lower than chance accuracy in the working memory capacity task with set size four (1 participant), (b) participants with more than 30% excluded trials in the reinforcement learning task (3 participants), and (c) participants who repeated the same response-key more than 80% of the trials (1 participant), in total 5 participants (age mean = 32, range 21–33; 3 males, 2 females) were excluded altogether.

## Results

Our main aim was to examine whether outcome-irrelevant learning changed as a function of individuals’ working memory capacity, and load manipulation (i.e., no-load, low, and high load). For this aim, we fitted a hierarchical Bayesian logistic regression. The dependent variable was the individuals’ tendency to repeat response key selection from the first to the second offer of the reinforcement learning bandit task (i.e., 0 vs. 1 for different or same, respectively; Stay_response key_). We examined three predictors including previous-outcome (i.e. £0 vs. £1, play pounds), working memory load (i.e., no-load, low load, and high load), individual’s working memory capacity, and their paired and triple interactions (see SI). Following recent guidelines on Bayesian regression analysis^39^, we start by performing nested model comparisons, allowing us to drop predictors that have a negligible contribution to the prediction of response key selection, and continue by examination of the posteriors of the best performing model. We assembled four nested models that included the following fixed effects: (a) Model 1 (full) previous-outcome (unrewarded vs. rewarded; describing outcome-irrelevant learning), working memory load manipulation, working memory capacity, and all paired and triple interaction as predictors. (b) Model 2 (working memory load) excluded working memory capacity parameters, (c) Model 3 (capacity) included working memory capacity parameters but excluded working memory load parameters, and finally, (d) Model 4 (null) included only previous-outcome as a predictor. Since we were interested in the population level (fixed effects) parameters, all models included a random effect of subjects on intercept, previous-outcome, working memory load, and previous-outcome x working memory load parameters. Leave-one-out cross-validation model comparison^40^ indicated that the best explanatory model was Model 3 (with previous-outcome, capacity, and their paired interaction). Specifically, stacking weights with uniform model priors showed 71% support for Model 3, 26% for Model 1, 3% for Model 2, and 0% for Model 4. We then continued to examine the posterior parameter’s distribution for the best fitting model, Model 3.

We found strong evidence for outcome-irrelevant learning, such that participants were more likely to stay with their response key selection after the first offer was rewarded (48%) vs. unrewarded (45%; posterior median = 0.09, HDI_95%_ between 0.03 and 0.16; probability of direction (pd) 99.75%; 0% in ROPE (− 0.013 − 0.013) and Bayes Factor (BF) of 7.66 against the null and of 7.29 against the modified ROPE; Fig. 2C). This replicates previous findings of outcome-irrelevant learning in human individuals^7–9^. Importantly, we found support for our pre-registered exploratory hypothesis that individuals with low working memory capacity will demonstrate increased outcome-irrelevant learning compared to high capacity individuals such that the interaction between previous-outcome and capacity was negative (posterior median = − 0.14, HDI_95%_ between − 0.22 and − 0.07; probability of direction (pd) 100%; 0% in ROPE (− 0.013 − 0.013) and Bayes Factor (BF) of 280 against the null and of 289 against the modified ROPE; Fig. 2E). Figure 2D illustrates a point-estimate of individual coefficients for previous-outcome (posterior mean) as a function of their estimated capacity. To provide the reader with a more intuitive effect-size of the association between working memory capacity and outcome-irrelevant learning, we calculated and found a correlation of r = − 0.31 (HDI95% between − 0.44 and − 0.17) between these two estimates. To affirm our results, we estimated working memory capacity using the change detection trials performed within the reinforcement learning task. We found that these estimates were positively correlated with working memory capacity estimates that were calculated using trials from the single change-detection task (r = 0.33, CI_95%_ between 0.2 and 0.45, BF_10_ = 4787). Importantly, we found working memory capacity estimates drawn from the reinforcement learning task, to also negatively correlate with outcome-irrelevant learning (r = − 0.26, CI_95%_ between − 0.38 and − 0.11, BF_10_ = 47). Thus, overall, we found similar findings regardless of whether working memory capacity was measured using behavior from the single or the dual task.

**Figure 3:**
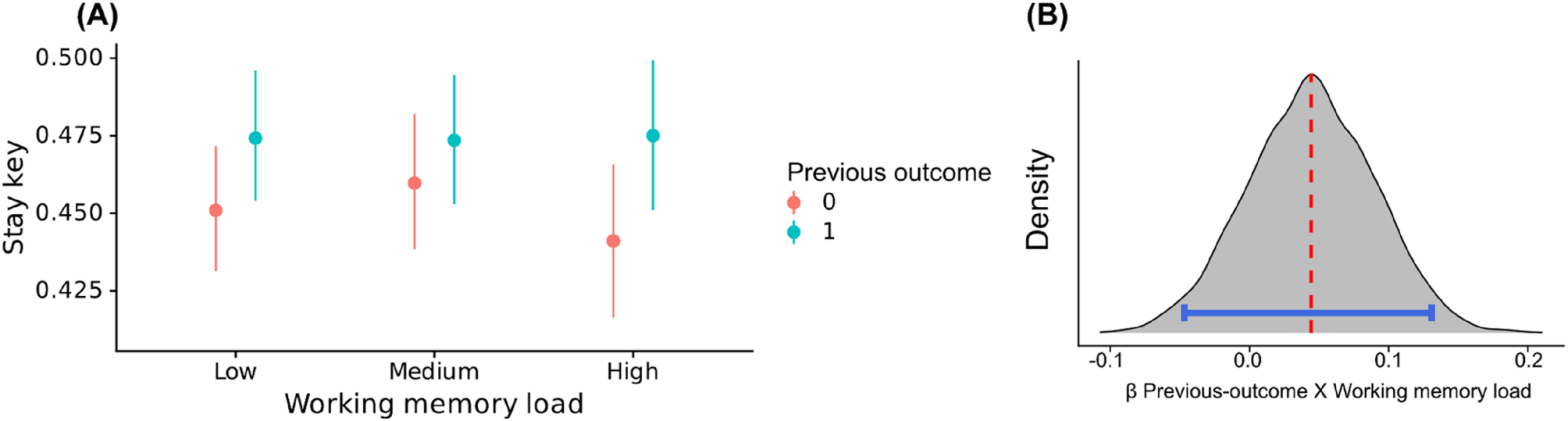
Outcome-irrelevant learning as a function of working memory load manipulation. (**A**) Descriptive results show that outcome-irrelevant learning was overall similar under all three load conditions. (**B**) The posterior distribution for the interaction regression coefficient suggested no concise support for or against the existence of a moderation effect (i.e., paired interaction between previous-outcome and working memory load; red line showing the posterior median and blue lines the HDI_95%_).

We did not find support for an effect of our working memory load manipulation on outcome-irrelevant learning as similar outcome-irrelevant learning was found under no load (2.2%) vs. high load (3.4%) (posterior median = 0.04, OR = 1.04). The HDI95% was between − 0.04 (OR = 0.96) and 0.12 (OR = 1.12) indicating that the most probable values of this interaction effect are small in magnitude^41^. Within these small effects, there is a probability of 84% of the effect being positive, and a probability of 16% of it being negligible (within the ROPE (between 0.013 and -0.013)). A BF analysis indicated similar ambiguity regarding the existence of the effect: there was no support for or against a non-zero effect (BF_01_ of 1.37) nor in favor of or against a non-negligible effect (BF_01_ = 1.42) (see Fig. 3).

To assert participants complied with our instructions and performed both the working memory and reinforcement learning components above chance, we examined accuracy rates. For the working memory task component, we found 94%, 88%, and 67% accuracy rates for no-load, low-load, and high-load, respectively. For the reinforcement learning task component, we found that participants were able to choose the card with the higher true reward probability in 57% of trials (no difference was found between different working memory loads; see SI for further details). Thus, we conclude that performance was well above random in all parts of the task (see SI for Bayesian analysis of accuracy rates). We conducted further analyses to examine whether the moderating effect of working memory capacity is specific to outcome-irrelevant learning or whether it has a more general effect on learning. First, we estimated accuracy rates in cards’ choices, with an accurate choice defined as selecting the card with a higher true reward probability. We then estimated and found that working memory capacity was not correlated with participants’ accuracy in choosing the more rewarding card in the pair (r = 0.08, CI_95%_ between − 0.07 and 0.22, BF_01_ = 3.2 in favor of the null). Furthermore, we repeated our main regression analysis while including learning accuracy as a covariate. We found that the moderating effect of working memory capacity on outcome-irrelevant learning remained substantial (posterior median = − 0.15, HDI_95%_ between − 0.24 and − 0.06; probability of direction (pd) 100%).

To sum up, the results demonstrated support for outcome-irrelevant learning at the group level and supported the moderation of working memory capacity on outcome-irrelevant learning estimates. However, our results did not support our hypothesis for interaction between working memory load and previous-outcome (see SI and Table S1 for further information regarding evidence in favor of the null hypothesis for working memory load manipulation effect).

## Discussion

To engage in goal-directed behavior, human agents must form action-outcome associations within the environment^42^. Reinforcement learning studies have described, in-depth, the mechanisms involved in assigning credit to preceding actions when inferring causal associations^43^. However, little is known regarding how cognitive control processes might prevent individuals from assigning credit to aspects of the environment that are known—with high certainty—to be irrelevant to the observed outcome^44^. To the best of our knowledge, this preregistered study was the first to demonstrate a moderating role for a well-known cognitive control system, namely working memory capacity^45^, on the tendency to assign value to irrelevant features of the environment^7–9^.

Our results replicated, at the group level, a newly described phenomenon coined “outcome-irrelevant learning,” which refers to a tendency to assign credit to outcome-irrelevant elements of a task^7–9^. Specifically, in the current study, participants completed a reinforcement learning task in which they were asked to select cards to gain rewards. Card selection was indicated by a keypress; importantly, only the cards’ visual features, but not the response key used for selection, predicted monetary outcome. Outcome-irrelevant learning was illustrated by an increased probability to stay with a response key selection when it was followed by a reward compared to an unrewarded outcome. Note that this result does not reflect a general tendency to repeat one’s previous actions, and thus should not be considered as an example of a perseveration tendency^46,47^. Importantly, outcome-irrelevant learning is different from perseveration as it is a reward-dependent effect, while response-key perseveration effects address a general tendency to repeat one’s choices regardless of reward or value. An individual showing a perseveration effect would tend to repeat their response-key choices regardless of the obtained reward. However, an outcome-irrelevant learner would show a lower/greater probability to repeat the previous response-key selection after unrewarded/rewarded trials, respectively. Thus, the main difference between perseveration and outcome-irrelevant learning is that the former is unrelated to reward, while the latter is. Note that both can co-occur in a single individual. In fact, when we predict response-key stay probability our intercept estimates correspond to overall response-key perseveration (tendency to stick/switch to a response key regardless of reward), while the effect of previous-outcome reflects outcome-irrelevant learning (increased/reduced tendency to stick with response key-selection following rewarded/unrewarded outcomes). In that sense, these effects (intercept and slope) are independent by design. Finally, an important aspect of outcome-irrelevant learning is that the effect was demonstrated despite both explicit instructions and practice trials indicating that the response keys were irrelevant to the monetary outcome. Therefore, unlike previous reinforcement learning studies that focused on an ability to learn which features are relevant to an outcome in an unknown environment^2,3,6,48^, outcome-irrelevant learning reflects a fundamental human difficulty to avoid assigning credit to features that are known, with high certainty, as not being causally related to obtaining a certain outcome^7–9^.

An important, and largely unanswered, question regards the underlying cognitive mechanisms of outcome-irrelevant learning. We argue that outcome-irrelevant learning reflects a control failure, whereby human agents find it difficult to avoid assigning credit to representations that are actively held in working memory^49–51^. In the current study, individuals were faced with duality, such that response keys are both task-relevant (thus, must be actively held in mind) and outcome-irrelevant (thus, should be disregarded and not be assigned value regardless of the outcome). One hypothesis for the mechanism allowing individuals to modulate outcome-irrelevant learning might be that control is exerted during value updating, thereby resulting in task-relevant, yet outcome-irrelevant, information to maintain a neutral value. Another possibility might be that response keys are assigned a value, but that cognitive control processes regulate their involvement during the decision-making process. Both explanations lead to the similar conclusion that cognitive resources are required for the mental shielding of outcome-relevant from irrelevant information during value updating. If outcome-irrelevant learning does indeed reflect a cognitive control failure, it should increase when attentional control resources are low.

Our main finding indicated that individuals with low, as compared to high, working memory processing capacity showed increased outcome-irrelevant learning. Working memory capacity is a well-studied phenomenon in the cognitive control literature, and it has been shown to predict individuals’ ability to avoid distraction from irrelevant information^20,21,25,32,52^. Individual differences in working memory capacity were previously found to predict interference from irrelevant information across multiple tasks such as the Stroop, dichotic listening, and anti-saccade tasks^16,22,24^. Our main finding extends this literature by demonstrating that working memory capacity also predicts value-based attentional control. This finding, although correlative in nature, is in line with the speculation that lower working memory capacity leads to difficulty in holding distinct representations of the task set, which consequently leads value to be assigned to representations that are activated at the time of selection. An alternative notion of working memory may focus on its role as short-term storage^45^. Accordingly, a potential contrary hypothesis relies on the idea that high working memory individuals are able to hold more information actively in mind, thus allowing them to encode a richer representation of their actions. In such a case, it might be that individuals with higher working memory capacity would show increased, rather than decreased outcome-irrelevant learning. However, our results show the opposite direction. It should be noted that, to date, most reinforcement learning studies have not directly addressed the influence of working memory capacity on credit assignment. Studies that have explored the role of working memory in credit assignment mostly manipulated set-size (e.g., number of arms in a multi-armed bandit task), thus primarily demonstrating an effect of decay and noise on the representations of relevant information^28–31^. Our findings suggest that working memory capacity, as measured by a visual working memory task, can predict the extent of inaccurate credit assignment.

The finding that working memory capacity, but not load manipulation, was associated with outcome-irrelevant learning should be discussed. One possibility might be that we did not include a strong enough load manipulation; however, the no-load (zero squares to remember) and the high-load (four squares to remember) conditions that we used should reflect the extreme ends of the load manipulation^53^. Another explanation might be that participants sacrificed effort on one task for the other, however, overall accuracy rates in both tasks were above chance, suggesting that participants did in fact devote attention and effort to both tasks. We, therefore, suggest a theoretical explanation for our finding, such that the visual stimuli (working memory task component) and the cards’ values (reinforcement learning task component) did not tap into the same working memory buffer, thus leading to the observed null effect. In this respect, the discrepancy in modalities might be the reason that loading working memory did not influence outcome-irrelevant learning. Specifically, we loaded working memory with visual information, while outcome-irrelevant information was modulated in the spatial-motor domain. Indeed, although these findings are still in debate, previous studies show that accuracy in a visual working memory task was more strongly disrupted by a visual rather than a spatial interference and vice versa^54–56^. Therefore, future studies are required to shed light on whether outcome-irrelevant learning is also more domain-specific than we assumed here. In a somewhat similar manner, and in line with the activity-silent memory theory^57–59^, information that is not immediately relevant for a present task is maintained with minimal neural activity^60^. Therefore, it could be that visual information from the working memory task component was maintained in activity-silent memory during the performance of the reinforcement learning task component^61^. Another reason for the failure to find an effect of working memory manipulation might be related to the way we defined working memory. Specifically, while we followed the definition of working memory as an active attentional system that filters irrelevant information^24^, several studies have also demonstrated dissociations between attention and working memory^62–65^. Therefore, it might be that the filtering process that is hypothesized to operate during credit assignment is associated with an attentional filtering process that is not directly controlled by the working memory system. In such a case, it is reasonable that working memory and attentional filtering abilities would correlate between individuals, which would lead to the observed association between working memory and outcome-irrelevant learning. However, this would also mean that working memory manipulation should not show a direct effect on outcome-irrelevant learning, which is also in line with the observed results.

Finally, we note that the outcome-irrelevant learning demonstrated in this study was primarily discussed in regard to the assignment of credit to response keys, but it can also be interpreted in terms of value assignment to spatial location (since the card in the right/left location was always selected using a right/left response key, respectively). The present study was designed to determine the effects of working memory capacity and load manipulation on credit assignment to outcome-irrelevant features of the environment. The results of this investigation showed that individual differences in working memory capacity predict outcome-irrelevant learning. However, it should be noted that we only examined individual differences in visual working memory, and it remains to be investigated whether these differences will also be apparent in different modules. We conclude that resource-demanding cognitive control is exerted during credit assignment, in such a way that credit can be accurately assigned to outcome-relevant actions. These findings contribute to our understanding of how credit assignment is regulated and provide a basis for future cognitive and clinical research.

## Supplementary Information

Supplementary Information.

## Data Availability

The data, analysis scripts, and the task can be found at the following address https://osf.io/rfeqx/. Preregistration on the open science framework (OSF) website is available at https://osf.io/6cz29.
