# Supplementary material for: Working memory capacity estimates moderate value learning for outcome-irrelevant features
Source: Sci Rep. 2022 Nov 16;12:19677. doi: 10.1038/s41598-022-21832-x (PMC9669000; doi:10.1038/s41598-022-21832-x)
Supplement: Supplementary file 1 — Supplementary Information. [file 41598_2022_21832_MOESM1_ESM.docx]

# **Supplementary Information**

### **Methods**

#### **Procedure:**

Participants were recruited using the prolific platform ([www.prolific.co](http://www.prolific.co)m). To avoid prolonged online testing in a single session, the experiment was separated into three sessions. In the first session, participants completed a working memory capacity task. In the second and third sessions, participants completed 150 trials of the reinforcement learning task (50 trials from each condition).

**Reinforcement learning task:**

The task was programmed using the jspsych package (v6.2.0) in JavaScript.

The task started with a memory array that had either no-load (0 squares), low-load (1 square), or high-load (4 squares; manipulated between blocks). The size of each square was 1.3° x 1.3° (all visual angles assume a 60cm distance of the participant from the screen) and the colors of the squares were randomly picked in each trial from 9 highly discriminable colors: red, magenta, blue, cyan, green, yellow, orange, brown, and black. (RGB values, respectively: 254,0,0; 255,0,254; 0,0,254; 0,255,255; 0,255,1;255,255,0; 255,128,65; 128,64,0; 0,0,0;). However, size and color may have varied as the experiment was conducted online. The no-load condition included a memory array with a square in a constant color throughout the entire block, thus not requiring subjects to withhold the color in working memory during the task. The low load condition included a memory array with one square in a varying color in each trial. The single square in those two conditions could have appeared in one out of six possible locations: Upper: (0,2.7°);(0,5.3°);(0,8°); Lower: (0,-2.7°); (0,-5.3°);(0,-8°). The high load condition included a memory array with four squares to remember. Each of the four squares appeared in a different quarter of the screen (Q1-Q4). Within each quarter, there were three possible locations in which a square could appear, resulting in a total of twelve possible locations: Q1: (2.7°,2.7°);( 5.3°,5.3°);( 8°,8°), Q2:(-2.7°,2.7°);( -5.3°,5.3°);( -8°,8°;), Q3: (-2.7°,-2.7°);( -5.3°,-5.3°); (-8°,-8°), Q4: (2.7°,-2.7°); (5.3°,-5.3°); (8°,-8°). After the memory array had disappeared, two cards appeared on the two sides of the screen ([see Figure S1](#_nqboxxruhv7o)). The two cards were randomly sampled out of four possible cards in each block. The cards were marked using black and white icons of daily objects ([see Figure S1](#_nqboxxruhv7o)). In each block, a new card deck was shown to the subjects. After choosing one of the two cards, participants probabilistically got a reward (gold coin and a writing ‘won a coin’) or did not get a reward (black circle and a writing ‘0 coins’) according to a random walk ([see Figure S2](#_hcgzzu3qz431)).

##### Verbal instructions:

***“****Welcome to the card game.* *Your winnings in this game will earn you additional payment bonus for the study. If no extra money will be earned in the card game, you will still get £2.5 for completing this session of the study. However, you can gain up to an extra £0.9 based on winnings in the game.*

*We will now provide instructions regarding the card game. Please read them carefully.*

*Feel free to go back and forth between the screens. At the end of the instructions, we will ask you to complete a short quiz about them, to make sure everything is well understood.*

*Below is an example of a card-deck of four cards, very much like the cards in the game to follow.*

*On each step, only two cards from the 4-card deck will be offered (as shown in the example below). You will be able to select the left card by pressing 's' and the right card by pressing 'k' on your keyboard. Please do your best to respond as fast and accurately as you can. After selecting the card, you will see an outcome in the middle of the screen, as shown below.*

*Winning very much depends on the card you chose - some cards are better than others in their deck. Your task is to find out which card is the best in each deck at any time and choose it. Please note that the winning chances of the cards are independent. Learning about one card does not tell you anything about the other cards.*

*Three important things to remember:*

1. ***How good a card is can change along with the game*** *- somewhat like the value of market products that are sometimes worth more and sometimes less.*
2. ***Only the cards are related to your chances of winning*** *- the location of the card and the response key you used to select it does not influence the chances of winning a coin.*
3. ***The chance that each card will give you money has nothing to do with the other cards*** *- you can't learn about one card from the money rewards you got for the other.”*

##### Verbal quiz

To ensure participants read and understood the task’s instruction they had to complete a verbal forced-choice quiz with 100% accuracy. Participants that made mistakes in the quiz, were prompted to the beginning of the instructions. The quiz questions were as follows (**correct answer in bold**):

1. *“What is the size of a card deck?” {2,****4****,6}*
2. *“How many cards are presented on each step?” {****2****,4,6}*
3. *“How do you choose a card?” {"Press the LEFT or RIGHT arrow keys",* ***"Press the ‘S’ or ‘K’ key with your LEFT or RIGHT hand."****, "Click on it"}*
4. *“How do you choose if a square is same or different?” {"Press the LEFT or RIGHT arrow keys",* ***"Press the ‘S’ or ‘K’ key with your LEFT or RIGHT hand."****, "Click on it"}*
5. *“How ‘good’ or ‘bad’ a card is will change along the game.” {****"True"****, "False"}*
6. *“What is the goal in the game?” {"The goal is to learn which card is better",* ***"The goal is to both remember the squares and learn which cards are bette****r****"****}*
7. *“If one card leads you to more money, it means the other cards will probably lead you to less money”. {"True",* ***"False"****}*
8. *“If you use the RIGHT and not the LEFT response keys, you might win more”. {"True - the response keys are related to the winning chances",* ***"False - you won't win more or less using RIGHT or LEFT response keys. Only the cards are related to your chance of winning."****}*

#### **Working memory capacity estimates:** The colors of the squares were randomly picked in each trial from 9 highly discriminable colors: red, magenta, blue, cyan, green, yellow, orange, brown, and black. (RGB values, respectively: 254,0,0; 255,0,254; 0,0,254; 0,255,255; 0,255,1;255,255,0; 255,128,65; 128,64,0; 0,0,0;) ([see Figure S3](#_mw6t5038h0qn)). The squares were equally distributed around the four quadrants of the screen. Within each quadrant, one (set size 4) or two (set size 8) square locations were randomly selected by the computer out of three possible locations in every quadrant of the screen. To take into account the fact that the experiment was made on participants’ personal computers, locations were on absolute distances compared to the center of their screen. The locations were 2.7°,5.3°, or 8° horizontally and vertically from the center of the screen, in each of the quadrants: Q1: (-2.7°,2.7°);( -5.3°,5.3°);( -8°,8°;) Q2:( 2.7°,2.7°);( 5.3°,5.3°);( 8°,8°) Q3: (2.7°,-2.7°); (5.3°,-5.3°); (8°,-8°) Q4: (-2.7°,-2.7°);( -5.3°,-5.3°); (-8°,-8°).

After data collection, we discovered that due to a technical error one location of the memory array squares on the lower left side of the screen was never probed during the retrieval phase of the change detection task. This could mean that participants learned that only 3/7 squares could be encoded during the 4/8 load conditions, effectively reducing the load on these conditions This issue occurred both in the single change detection task used to estimate the working memory capacity (set-size of 4/8) and the high load condition of the dual-task (set-size of 4). Since individuals might have noticed that locations from one quarter are never probed, they could have encoded less information, effectively holding in mind 7 and 3 squares, in the 8 and 4 squares load conditions, respectively. We were concerned that this reduced load might have lowered the observed effect of working memory load manipulation on outcome-irrelevant learning. To ensure that this issue did not affect our overall conclusion, we repeated our main analysis, predicting response-key stay probability with working memory load and previous-outcome, only for the first 25 trials of the dual-task, where participants were less likely to notice that squares from a specific location were never probed as a target. We found similar results to the one reported in the main text so that with no substantial difference in outcome-irrelevant learning for high vs. low-load conditions (posterior median =.03, HDI95% between -.18 and .22; probability of direction (pd) 61%). Thus, our overall conclusion should not be affected by the occurrence of this technical error.

### **OSF project**

The data, analysis scripts, and the task can be found at the following address <https://osf.io/rfeqx/>.

Preregistration on the open science framework (OSF) website is available at <https://osf.io/6cz29>.

### **Results**

##### **Regression analysis:** For our analysis, we dummy-coded the outcome in the first offer to be a binary variable (i.e., 0 vs. 1 for *rewarded* or *unrewarded*), and the load condition was also dummy-coded (i.e., no-load, low load, and high load). Finally, we used the centered average working memory capacity.

##### **Priors robustness analysis:** We used our pre-registered priors (N(0,0.2) for the reward main effect and N(0,0.09) for the interaction effects). We conducted a prior robustness analysis showing that reported effects hold also when having narrower (N(0,0.09) or wider (N(0,0.4) priors for our predictors ([see Figure S4](#_mtrdfe51jfsp) for an illustration of the prior of the previous-outcome main effect).

##### **Prior and posterior predictive checks:** We performed prior and posterior predictive checks to assess the suitability of our priors to the empirical results. We found good correspondence between the predicted samples and empirical choices ([Figure S5](#_h1jlgd3nfiyv)).

##### **Accuracy estimates:** To assert participants complied with our instructions and performed both the working memory and reinforcement learning components above chance, we aimed to show that accuracy rates were above chance in both tasks. Specifically, we performed a hierarchical bayesian logistic regression with an intercept predicting the accuracy of each trial. We found in the reinforcement learning task that participants had picked the more rewarding card in most of the trials (57%; Intercept posterior median 0.32 with HDI_95%_ between 0.27 and 0.38 (estimates are in log-odds so that zero refers to chance level). Furthermore, no difference in accuracy was found between working memory loads with 58% accuracy in baseline and a similar accuracy for the low-load condition (57%; posterior median -0.02 with HDI_95%_ between -0.08 and 0.03) Similarly, high-load also had no difference in accuracy performance (57%; posterior median -0.03 with HDI_95%_ between -0.09 and 0.03). As for the working memory task component, we found 94% accuracy rates for no-load (posterior median = 3.18, HDI_95%_ between 2.99 and 3.36), 88% for medium load (posterior median = 2.21, HDI_95%_ between 2.07 and 2.34, and 67% for high load (posterior median = 0.74, HDI_95%_ between 0.65 and 0.83. Thus, we conclude that performance was well beyond random in all parts of the task.

| ***Table S1.*** *Saturated model (Model 4) parameters estimation including previous-outcome, working memory*  *load, and working memory capacity as predictors for Stay_key-response_.* | | | | |
| --- | --- | --- | --- | --- |
| **Parameter** | **Median** | **95%CI** | **pd** | **%In ROPE [-0.013 - 0.013]** |
| Intercept | -0.20 | [-0.29, -0.11] | 100% | 0% |
| Reward_first_offer_ | 0.09 | [0.02, 0.17] | 99.3% | 0% |
| Capacity | 0.04 | [-0.06, 0.13] | 78.9% | 16% |
| Set size medium | 0.03 | [-0.03, 0.09] | 84.4% | 20% |
| Set size high | -0.04 | [-0.12, 0.03] | 84.9% | 17% |
| Reward_first_offer_ x Capacity | -0.12 | [-0.20, -0.04] | 100% | 0% |
| Reward_first_offer_ x Set size medium | -0.04 | [-0.11, 0.04] | 82% | 18% |
| Reward_first_offer_ x Set size high | 0.04 | [-0.04, 0.12] | 84% | 16% |
| Capacity x Set size medium | 0.01 | [-0.07, 0.10] | 61% | 23% |
| Capacity x Set size high | 0.07 | [-0.03, 0.16] | 92.6% | 9% |
| Reward_first_offer_ x Set size medium x Capacity | -0.04 | [-0.13, 0.05] | 81% | 16% |
| Reward_first_offer_ x Set size high x Capacity | -0.05 | [-0.14, 0.05] | 86% | 13% |
| *Output from analysis with bayestestR R package [1].* | | | | |

#### **
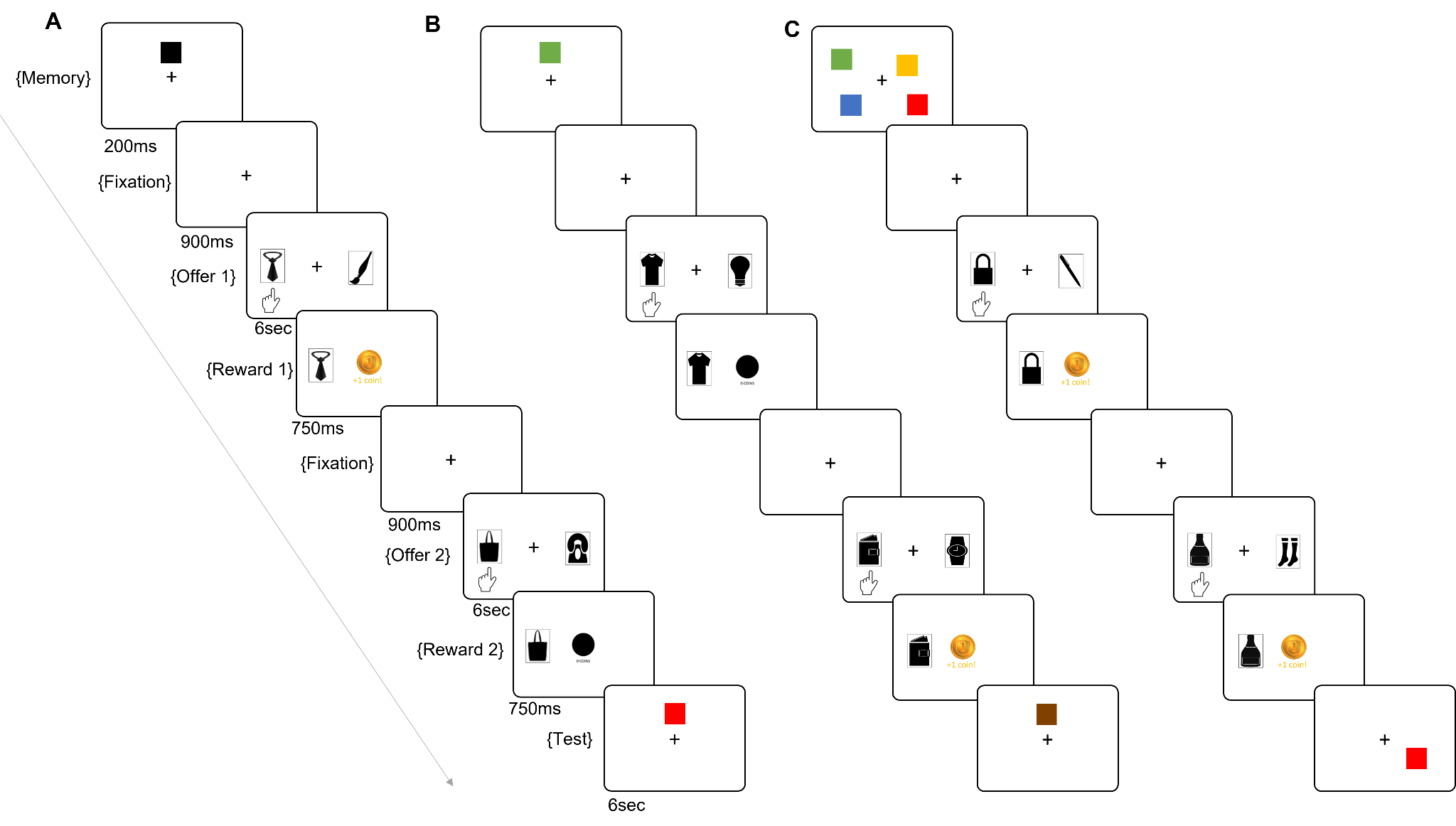
**

##### **Figure S1.** Trial sequence showing the reinforcement learning task. Participants were asked to first memorize a visual array (memory array stage), make two choices across two offers (reinforcement learning bandit task stage), and then report whether a target was the same or different compared to the visual array that was memorized at the trial initiation (test array stage). The task included three load conditions: **(A)** no working memory load, one square in the memory array phase having the same fixed color throughout the whole block, **(B)** low working memory load where one square appeared in a different color on each trial in the memory array phase, **(C)** high working memory load where four colored squares appeared in the memory array. The task allowed us to examine learning processes as a function of working memory load.

#####
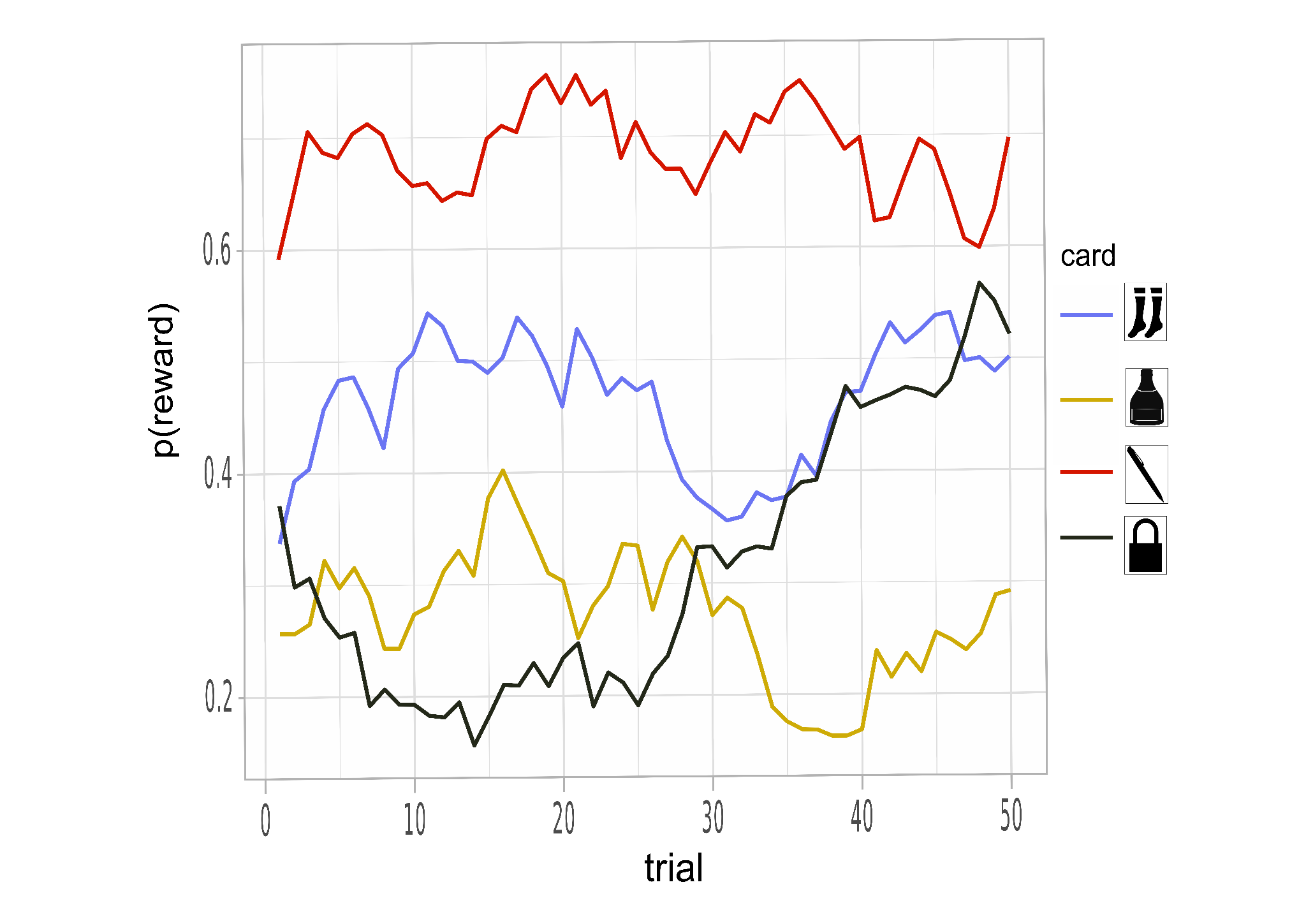


#####

#####

#####

#####

#####

#####

##### **Figure S2.** Reward probabilities for each card as a function of trial and card. The probabilities were stochastic to ensure participants keep on learning throughout the experiment [2].

**
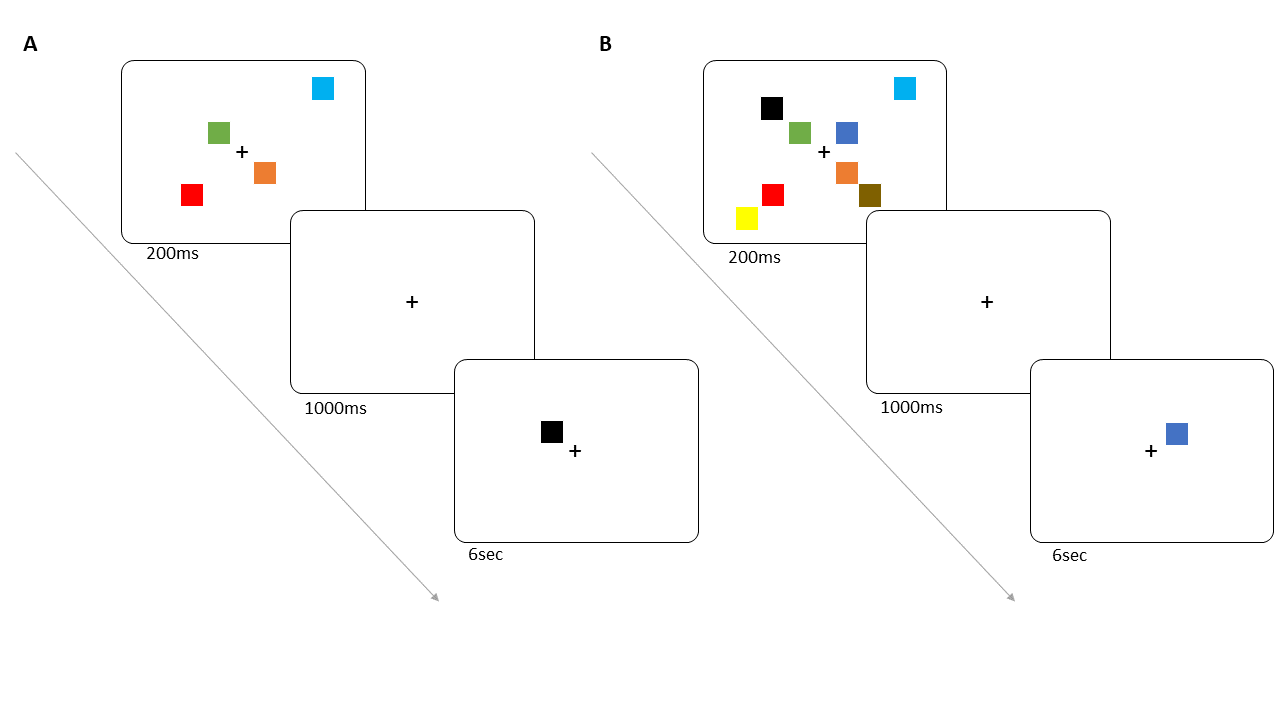
**

##### **Figure S3.** Trial sequence in the visual working memory task. To measure working memory capacity participants were asked to memorize a visual array, and then to retrieve it in the test phase to report whether a target square had the same or different color as the square that appeared in the same location. **(A)** the low load included four to-be-memorized stimuli and one target square. **(B)** the high load included eight to-be-memorized stimuli and one target square. Calculating the accuracy in the test phase allowed us to measure each individual’s working memory capacity.

#####

**
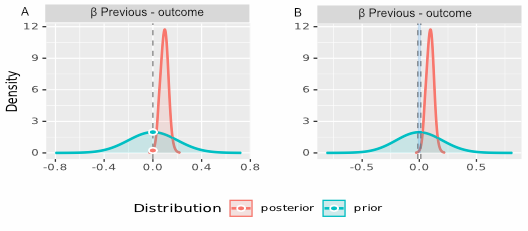
**

##### **Figure S4.** Depiction of the calculated Bayes Factor (BF) against the point-null and against the ROPE interval, using the posterior and prior distributions of our previous-outcome main effect in Model 2 (containing the previous-outcome and working memory capacity as predictors). **(A)** Savage-Dickey density ratio was calculated to approximate the BF against the point-null, showing that a null result is less likely given the data, and **(B)** BF against the ROPE was calculated using an approximation of the odds of a result within the ROPE interval (-0.013 - 0.013) given the prior or given the posterior.

**
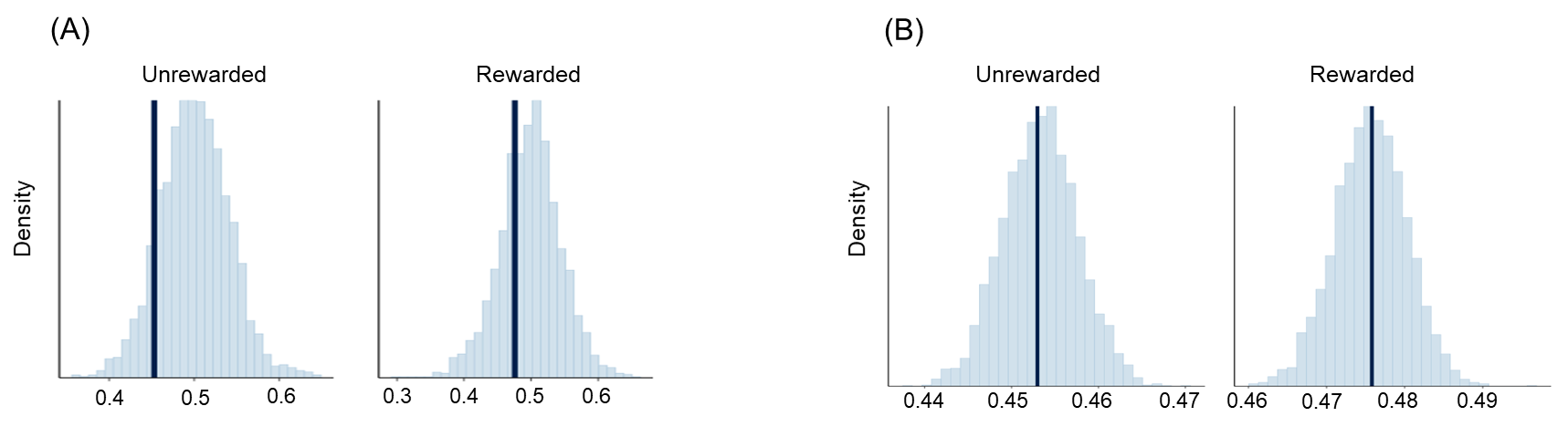
**

##### **Figure S5.** Prior and posterior predictive checks were conducted to reassure our ability to adequately predict the dependent variable. MCMC mean estimates are shown in pale blue and empirical data mean estimates are shown in dark blue. **(A)** Prior predictive checks disregard the data and thus show that both MCMC distributions are centered around a 0.5 probability. **(B)** Posterior predictive checks use a Bayesian model taking the data into consideration and thus MCMC predictions are different for rewarded vs. unrewarded. Overall, we found good correspondence between our prior and posterior estimates and our dependent variable.

# **References**

1. Makowski, D., Ben-Shachar, M. S. & Lüdecke, D. bayestestR: Describing Effects and their Uncertainty, Existence and Significance within the Bayesian Framework. *J. Open Source Softw.* **4**, 1541 (2019).

2. Delgado, M. R., Phelps, E. A. & Robbins, T. W. *Decision Making, Affect, and Learning: Attention and Performance XXIII*. (OUP Oxford, 2011).

##### 
